# Telocytes and Other Interstitial Cells: From Structure to Function

**DOI:** 10.3390/ijms22105271

**Published:** 2021-05-17

**Authors:** Sanda Maria Crețoiu

**Affiliations:** Department of Cell and Molecular Biology and Histology, Carol Davila University of Medicine and Pharmacy, 8 Eroii Sanitari Blvd., 050474 Bucharest, Romania; sanda@cretoiu.ro

The Special Issue, “Telocytes and Other Interstitial Cells: From Structure to Function” of the International Journal of Molecular Sciences, is dedicated to recent progress in research on interstitial cells. In general, by interstitial cells, one can understand the totality of cells found in the interstitium, the contiguous fluid-filled space that encompasses the places between the epithelial, muscular, and nervous tissues. In this space, telocytes are considered a relatively new and special type of interstitial cells, in other words, they were discovered more than 15 years ago (2005) by the team in which I am part of, at that time under the leadership of regretted Professor Popescu. Initially called interstitial Cajal-like cells, due to the similarity with interstitial cells of Cajal, they were then renamed telocytes in 2010, by consensus between their discoverer, Professor Popescu, and the one who is considered the greatest expert in the field of interstitial cells of Cajal, Professor Faussonne-Pellegrini [1].

A comprehensive review written by Maria Giuliana Vannucchi is dedicated to the telocytes located in the gut, with an accent on their morphology and immunophenotype, showing that even if telocytes are subcatgorized into subtypes, they are indeed the same cell [2]. The paper underlines once again the importance of Transmission Electron Microscopy (TEM) as the gold standard method for the identification of telocytes, underlining the importance of PDGFRα labeling for the recognition of the network formed by telocytes under the cryptal intestinal epithelium. The 3D networks are essential in performing the hypothetical functions played by telocytes in the gut: mechanical support during gut movements, compartmentalizing the stroma, and entrapping the other interstitial cells (macrophages, mast cells, fibroblasts, etc.) to which they exchange signals by means of stromal synapses or by extracellular vesicles [2]. Moreover, the role of telocytes in the cooperation with stem cells is also discussed, without overpassing the fact that they can even be precursors of the interstitial cells of Cajal [2].

Her contribution is completed by a complementary review by Foong and al. describing the role of interstitial cells of Cajal in gastrointestinal motility [3]. Interstitial cells of Cajal are known to act like pace-makers cells, and several subtypes are described in both murine species and humans. The importance of interstitial cells of Cajal is also debated in many gastrointestinal motility disorders, and the main issue is that most studies were carried out on animal models, and one needs to elucidate if the findings can be extended to humans [3]. Additional molecular studies are needed to characterize these cells from a transcriptomic, proteomic, and electrophysiological point of view, and this involves finding a reliable source of human interstitial cells of Cajal [3].

Bladder dysfunction might be attributed to suburothelial interstitial cells, which can act as connecting devices between the urothelium and detrusor muscle. The original paper of Neuhaus et al., describing the mechanosensitivity of suburothelial interstial cells from the human bladder focused on calcium transients detected in cultivated cells stimulated by various mechanical stimuli [4]. The authors propose a mechanism of non-neuronal signaling in the human bladder intermediated by a network of interstitial mesenchymal cells that occupy a strategic position between the urothelial layer and the deep lamina propria and express specific receptors and connexin Cx43, leading to the formation of a functional syncytium [4].

Telocytes were shown to express CD34 as a surface marker, and PDGFRα was also reported to be frequently co-expressed. Original contribution in this direction is brought to this Special Issue by the paper of Lis et al., demonstrating the spatial organization of CD34/PDGFRα double-positive cells in the human aortic valve [5]. Different morphology and spatial distribution of telocytes were shown in this paper using confocal microscopy. The distribution was different in the valve layers and is variable with age, evolving over a lifetime, suggesting that CD34/PDGFRα are important for maintaining local milieu resistant to calcification and implicitly to pathologic remodeling [5].

A research article conducted by Romano et al., on human primary cell cultures of skin telocytes describes a microbead-based method for the isolation of telocytes depending on the above-mentioned CD34/PDGFRα markers and aims to detect a reliable method for future in vitro investigations [6]. This in vitro study describes a novel two-step methodology using immunomagnetic microbeads to differentiate telocytes (CD31−/CD34+/PDGFRα+/vimentin+) from ECs (CD31+/CD34+/PDGFRα−/vimentin+) and fibroblasts (CD31−/CD34−/PDGFRα+/vimentin+) [6].

Since this Special Issue is also devoted to the other kinds of interstitial cells, Duliban et al., contributed with an original paper regarding interstitial Leydig cells as a starting point for tumors in human testis and their correlations with lipid homeostasis [7]. The paper, which addresses the connection between estrogens and leptin and adiponectin concentrations, revealed for the first time that aromatase and phospholipase C and other kinases can be part of adipokine signaling pathways in human Leydig cell tumors [7].

Gandahi et al., original contribution showed the presence of telocytes in the pancreas of Chinese soft-shelled turtles and suggested a role in intercellular communication [8]. The authors showed that amphibian telocytes have a classical similarity in ultrastructure to mammals, also displaying identical immunohistochemical characteristics co-expressing CD34+/Vimentin+ in the turtle pancreas [8].

Cretoiu D et al., described a very original perspective based on the simulation of a mathematical model of telocytes in culture relative to their involvement in intercellular communication phenomena [9]. Data obtained from ultrastructural images and video recordings of cells in culture are incorporated in special software to create predictive multiscale models as a foundation for an integrative, systems-level approach. The model created will create the opportunity to observe the involvement of telocytes in intercellular signaling and tissue regeneration. By creating software-based telocytes models in the future it will become easy to identify, simulate, and potentially optimize therapeutic means for the use of telocytes [9].

Another review is pointing to the role of telocytes in the pathology of white adipose tissue [10]. Telocytes and/or CD34+ stromal cells were analyzed in normal adipose tissue, and their distribution was compared with pathological processes found in adipose tissue, e.g., signet ring carcinoma with Krukenberg tumor, nevus lipomatosus superficialis of Hoffman–Zurhelle, amyloidosis, neuromas, and many other pathological circumstances [10].

To conclude the Special Issue and to form an opinion for the scientific community on the controversial role of telocytes and specially to see them integrated into the context of the concept of interstitial cells, there is one review dedicated to the telocytes found in the peripheral system [11]. Telocytes were described in nerves, sensory nerve endings, ganglia, and the intestinal autonomic nervous system, as well as in tumors of the peripheral nervous system. Many of their possible functions were discussed as well [11].

To summarize, I hope that this Special Issue composed of six original papers and four reviews further consolidates the interest of people studying the importance of interstitial cells/space and will open new avenues for future therapeutic approaches.
